# Surgical Management of High-Grade Meningiomas

**DOI:** 10.3390/cancers16111978

**Published:** 2024-05-23

**Authors:** Mark A. Pacult, Colin J. Przybylowski, Shaan M. Raza, Franco DeMonte

**Affiliations:** 1 Department of Neurosurgery, Barrow Neurological Institute, St. Joseph’s Hospital and Medical Center, Phoenix, AZ 85013, USA; mark.pacult@barrowneurosurgery.org; 2 Division of Neurosurgery, Fukushima Brain Tumor Center, Raleigh Neurosurgical Clinic, Raleigh, NC 27609, USA; cprzybylowski@raleighneurosurgical.com; 3 Department of Neurosurgery, The University of Texas M.D. Anderson Cancer Center, Houston, TX 77030, USA; smraza@mdanderson.org

**Keywords:** meningioma, atypical, anaplastic, extent of resection, gross total resection, Simpson grade

## Abstract

**Simple Summary:**

Meningiomas are classified pathologically by the World Health Organization (WHO) grading system, with WHO grade 2 and 3 tumors considered “high-grade”. These tumors are characterized microscopically by high mitotic rates and macroscopically by a brain invasion. The goal of the surgical treatment of these tumors is maximal safe resection to minimize surgical morbidity and extend patient survival. Surgical resection is made difficult by frequently indistinct boundaries between normal brain layers and the encasement of blood vessels and cranial nerves. Surgeons and scientists must continue to collaborate to offer patients the highest quality operative and post-operative medical care.

**Abstract:**

Maximal resection with the preservation of neurological function are the mainstays of the surgical management of high-grade meningiomas. Surgical morbidity is strongly associated with tumor size, location, and invasiveness, whereas patient survival is strongly associated with the extent of resection, tumor biology, and patient health. A versatile microsurgical skill set combined with a cogent multimodality treatment plan is critical in order to achieve optimal patient outcomes. Continued refinement in surgical techniques in conjunction with directed radiotherapeutic and medical therapies will define future treatment.

## 1. Introduction

Safe and thorough surgical resection is the primary treatment for patients with high-grade meningiomas. As endorsed by Simpson’s historic scale, this includes gross-total tumor resection (GTR) and the resection of the dura of origin [1]. In high-grade meningiomas, this decreases the rate of tumor recurrence and leads to lower all-cause mortality [2]. High-grade meningiomas, classified as grade 2 (atypical) and grade 3 (anaplastic) by the World Health Organization (WHO), can be challenging lesions to thoroughly resect, however [3]. Pial invasion and ill-defined arachnoid planes often increase the risk of injury to the cortex, cranial nerves, and eloquent vessels. The potential benefit of a complete resection on patient survival must be tactfully weighed against the detrimental effect of neurological morbidity on patient quality of life.

Here, we review the surgical management of high-grade meningiomas, with an emphasis on clinical outcomes. To date, clinical outcome data are composed solely of retrospective grade III evidence. A complex interplay of anatomical, clinical, and biological factors influences surgical and clinical results.

## 2. Perioperative Considerations

Patients who require a surgical resection of a presumed meningioma should undergo a thorough preoperative workup, including appropriate imaging studies and medical clearance for surgery. High-quality magnetic resonance tomography (MRI) images are critical in preoperative planning and should be available for synchronization to intraoperative neuro-navigation software. Functional MRI or diffusion tensor imaging (DTI) are not essential in presumed extra-axial lesions but may be considered to delineate the relationship of the tumor to eloquent structures, especially if brain invasion is suspected. Computed tomography (CT) is frequently obtained as part of the initial workup for suspected intracranial pathology. These images may help identify the relationship of the tumor to nearby bony anatomy and are essential in the preoperative planning of skull base lesions.

Meningiomas display characteristic imaging features, such as avid contrast enhancement and a dural “tail” demonstrating a dural attachment on T1-weighted contrast enhanced MRI. There are several suggestive radiographic features that may be used to differentiate high-grade from benign meningiomas, including a lower apparent diffusion coefficient (reflecting higher cellularity), the radiographic sign of “mushrooming”, and high signal intensity on fluid-attenuated inversion recovery (FLAIR) sequences. A definitive pathological grade, however, cannot be established radiographically [4,5].

A growing body of literature examines the use of radiomics and deep-learning models to predict meningioma grading from preoperative MRIs compared to proven histopathological grading [6,7]. These models identify certain features in pathologically proven high-grade meningiomas and apply these to test subjects. In a cohort of 181 patients, Zhu and colleagues applied a deep learning algorithm to identify the meningioma grade based on preoperative MRI with a sensitivity of 76.9% and specificity of 89.8% [7]. Such algorithms may become more prevalent and may be of use in guiding operative strategy based on presumed preoperative pathological grade, though at present they are not routinely applied.

## 3. Surgical Strategy and Technical Considerations

Meningiomas are approached surgically with classic operative tenets [8]. Because pathology cannot be determined preoperatively, the surgical strategy for meningiomas is generally consistent, regardless of the presumptive WHO grade.

### 3.1. Expose

Patient positioning, incision planning, and bone removal are planned to optimize surgical exposure. Techniques to relax the brain and obviate the need for brain retraction are emphasized. These include directed neuro-anesthetic management, thoughtful head positioning to maximize venous outflow, the manipulation of non-elegant tissues (skin, muscle, and cranium), the exploitation of natural anatomical corridors, and the early release of cerebrospinal fluid (CSF).

### 3.2. Devascularize and Debulk

The coagulation and resection of meningeal tumors from their dural attachment, which contains their primary bloody supply, is performed as soon as possible in the operation. A devascularized tumor is significantly easier to manipulate and dissect for the remainder of the resection. Intratumoral debulking is often necessary to decrease the mass effect for a better visualization of critical structures prior to circumferential dissection.

### 3.3. Dissect

The tumor/pia-arachnoidal plane is diligently identified and every attempt is made to remain extra-pial during the tumor resection. This may be particularly challenging and at times impossible with higher-grade meningiomas that have a propensity to invade the pia-arachnoid layers and the surrounding brain. Novel techniques, such as the use of intraoperative ultrasound (ioUS) elastosonography, have been reported in order to aid in the detection of the meningioma–brain interface and may be particularly useful in cases of high-grade meningiomas when this border is indeterminate. Della Pepa and colleagues report their experience with 36 patients utilizing this technique and found higher accuracy with ioUS elastosonography in predicting the brain–meningioma interface than preoperative MRI [9].

Techniques to definitively identify the meningioma grade intraoperatively may be useful in order to maximize the extent of resection and decrease the recurrence rate. Intraoperative flow cytometry has been reported to correctly identify the meningioma grade with 90.2% sensitivity and 72.2% specificity in an intraoperative sample of 59 patients reported by Alexiou and colleagues [10].

### 3.4. Coagulation and Resection of the Dura

The coagulation (and resection when possible) of the dural attachment is performed to decrease the risk of tumor recurrence. Tumors located along the cerebral convexity lend themselves to circumferential dural resection; tumors along the skull base or foramen magnum may only allow for dural coagulation.

## 4. WHO Grade 2 Meningiomas

### 4.1. Pathologic Features

The classification scheme for meningiomas is based on an analysis of histopathological features, such as degree of brain invasion, the number of mitotic figures (4 to 19 mitoses per 10 high-powered fields), cellularity, and cellular and regional architecture [11]. A major distinguishing factor separating grade 2 from grade 1 tumors is the degree of brain invasion. In addition to histopathological features, the latest WHO guidelines introduce molecular markers for certain meningioma subtypes, the most clinically important being the NF2 allele mutations and deletions of chromosome 22q [3,11]. Before the year 2000, such meningiomas comprised roughly 5% of all meningiomas; they now constitute up to 35% of all meningiomas [12]. Because the pathological definition influences postoperative treatment paradigms, such as radiation therapy, delineating surgical outcomes for this unique pathologic grade is critically important.

### 4.2. Surgical Outcomes and Survival

Survival and clinical outcome data for WHO grade 2 tumors are heterogenous. The literature is confined to retrospective studies, and the majority are single-institution series. A synopsis of selected studies with relevant data points is presented in Table 1.

**Table 1:** Selected studies examining WHO grade 2 or atypical meningiomas.

Authors	Year	Study Type	Setting	Number of Patients	Mean Follow-Up (Years)	GTR (%)	Recurrence Rate (%)	PFS (Years)	PFS (%)			OS (Years)	OS (%)			Factors Associated with OS ^iv^	Factors Associated with PFS ^iv^
									3 Years	5 Years	10 Years		3 Years	5 Years	10 Years		
Aizer et al. [2] ^i^	2015	Retrospective	National Cancer Institute Database	575	3.9	47.3								91.3 (GTR) 78.2 (STR)		Improved: GTR	NA
Kumar et al. [13] ^i^	2015	Retrospective	Single institution	22	3.7	41.0				58%				83%		NA	NA
Soni et al. [14]	2021	Retrospective	Single institution	214	4.5	73.8	31.8	1.9		70.7 (GTR) 40.5 (STR)	60.6 (GTR) 25.5 (STR)	4.2		87.8 (GTR) 78.4 (STR)	83.0 (GTR) 43.1 (STR)	Improved: GTR	Improved: GTR
Goyal et al. [15]	2000	Retrospective	Single institution	22	5.5	68.2	36.4	3.8				10.6		91	76	None	None
Rydzewski et al. [16] ^i^	2018	Retrospective	National Cancer Database	7811		25.1							89.3	75.9		Improved: GTR without RT, RT Worse: age > 50, non-Hispanic black race, comorbidity score ≥ 2, community hospital setting	NA
Palma et al. [17]	1997	Retrospective	Single institution	42		100	52 (5 year)	11.9		77	55			95	79	Improved: Simpson I	NA
Durand et al. [18] ^i^	2009	Retrospective	Multi institution	166	5.4	92.2				48.4	22.6			78.4	53.3	Improved: Simpson I, age < 60 years ^iii^	Improved: No RT
Yang et al. [19] ^i^	2008	Retrospective	Single institution	40	5.3	85	10	11.5			87.1	11.8			89.6	None	Worse: EOR
Gabeau-Lacet et al. [20]	2009	Retrospective	Single institution	47	5.5		27.7	4.7	65	48		13.2		86	61	Worse: bone involvement	Worse: bone involvement
Jo et al. [21]	2010	Retrospective	Single institution	35	3.3	31.4 ^ii^	0 (GTR) 32 (STR)	2.1 (GTR) 2.2 (STR)								NA	NA
Moon et al. [22] ^i^	2012	Retrospective	Single institution	55	3.8	50.9	25.5	3.6								NA	GTR
Hardesty et al. [23]	2013	Retrospective	Single institution	228	4.3	58	22	1.7								NA	NA
Park et al. [24]	2013	Retrospective	Single institution	83	3.6	66.3	44.6	2.1		48	48		90.2	62		NA	Improved: RT, GTR
Zaher et al. [25]	2013	Retrospective	Single institution	44		36.4	36.4	3.3				4.8		35		Improved: GTR, age < 50 years	Improved: RT
Pasquier et al. [26] ^i^	2008	Retrospective	Multi institution	82		71 ^ii^		2.1		62				67.5		Worse: Age > 60 years, high mitotic rate ^iii^	Worse: high mitotic rate ^iii^
Hammouche et al. [27]	2014	Retrospective	Single institution	79	4.2	43 ^ii^	30	5.5		53		10.7		81		NA	Worse: higher Simpson grade resection
Wang et al. [28]	2015	Retrospective	Single institution	28	4.8	50	46.4	5.3						100		NA	Improved: GTR, MIB-1 < 8%
Da Broi et al. [29]	2021	Retrospective	Single institution	77	5.5	70.1	28.6		64.9	51.9	20.8		86.3	81.9	65.7	Improved: Age < 65, preoperative KPS ≥ 70, no retreatment (surgery or RT)	Improved: GTR

^i^ Study included anaplastic meningiomas in cohort, data presented separately in Table 2 (patient numbers listed separately); ^ii^ Defined GTR as Simpson I only; ^iii^ Data point refers to atypical and anaplastic meningiomas; ^iv^ Statistically significant associations in multivariate models with *p* < 0.05 only. NA, not applicable.

**Table 2:** Studies examining WHO grade 3, anaplastic, or malignant meningiomas.

Authors	Year	Study Type	Setting	Number of Patients	Mean Follow-Up (Years)	GTR %	Recurrence Rate (%)	PFS (Years)	PFS (%)			OS (Years)	OS (%)				Factors Associated with OS ^iv^	Factors Associated with PFS ^iv^
									3 Year	5 Year	10 Year		2 Year	3 Year	5 Year	10 Year		
Moliterno et al. [30]	2015	Retrospective	Single institution	37	2.6 years	59						2.7	66.6		27.9		None	NA
Kumar et al. [13] ^i^	2015	Retrospective	Single institution	15	3.7 years	33.3				20					23		NA	NA
Rydzewski et al. [16] ^i^	2018	Retrospective	National Cancer Database	1936		15.7							70.9		55.4		Worse: Age > 50	NA
Aizer et al. [31] ^i^	2015	Retrospective	National Cancer Institute Database	64	3.9	54.7									64.5 (GTR) 41.1 (STR)		Improved: GTR	NA
Orton et al. [32]	2017	Retrospective	National Cancer Institute Database	755		58									41.4		Improved: RT Worse: older age, higher comorbidity score, STR	NA
Peyre et al. [33]	2018	Retrospective	Multi institution	57	4.8	75		2.3				2.6	84		10	10	Improved: De novo anaplastic status, lower mitotic index	NA
Sughrue et al. [34]	2010	Retrospective	Single institution	63	5	63.5	47					4.2 (GTR) 8.9 (STR)	82		61	40	Improved: STR	NA
Champeaux and Jecko [35]	2016	Retrospective	Single institution	43	7.4	71.4						4.1	81.4		48.8	27.5	Improved: Mitosis count ≤ 14 per 10 HPF Worse: prior meningioma surgery	NA
Champeaux et al. [36]	2019	Retrospective	Multi institution	178	4.5	66.3						2.9			40	27.9	Improved: Age < 65, higher EOR, RT Worse: prior meningioma surgery	NA
Dziuk et al. [37] ^ii^	1998	Retrospective	Single institution	38			62.8		74	25							Improved: GTR, RT, de novo status	NA
Palma et al. [17]	1997	Retrospective	Single institution	29		100	84% (5 year)	2	45	15		6.89			64.3	34.5	Improved: Convexity location	NA
Durand et al. [18] ^i^	2009	Retrospective	Multi institution	33	5.4	90.9				8.4	0				44	14.2	Improved: Simpson I, age < 60 years, histological grade 2 ^iii^	None
Yang et al. [19] ^i^	2008	Retrospective	Single institution	24	3.5	66.7	75	2.7		29		3.3		55	35		Improved: RT Worse: Brain invasion, malignant progression, EOR, p53 overexpression	Improved: RT Worse: brain invasion, malignant progression, EOR, p53 overexpression
Pasquier et al. [26] ^i^	2008	Retrospective	Multi institution	37		71 ^v^		2.1 ^iii^		48					60		Worse: Age > 60 years, high mitotic rate ^iii^	Worse: High mitotic rate ^iii^
Tosefsky et al. [38]	2023	Retrospective	Multi institution	103	3.8	60	73%	3.2		37					66		Improved: RT, tumor necrosis Worse: Age ≥ 65 years, male sex, high N/C ratio	Improved: hypercellularity Worse: Age ≥ 65 years, male sex

^i^ Study included atypical meningiomas in cohort, data presented separately in Table 1 (patient numbers listed separately); ^ii^ Study included all malignant meningiomas; ^iii^ Data point refers to atypical and anaplastic meningiomas; ^iv^ Statistically significant associations in multivariate models with *p* < 0.05 only; ^v^ Defined GTR as Simpson I only. NA, not applicable.

Overall, GTR rates for WHO grade 2 meningiomas range widely among studies (25–100%) [16,17]. This likely reflects heterogeneity in a variety of factors, including patient population, tumor location, tumor size, individual surgeon skill, and the definition of GTR (some authors consider only a Simpson I resection to be GTR, whereas others include Simpson grades I–III in the definition). The radiographic recurrence rate in WHO grade 2 tumors ranges in the literature from 10% over a mean follow-up of 5.3 years to 52% at 5 years [17,19]. Overall survival (OS) and progression-free survival (PFS) rates at ten years range from 61–89.6% to 20.8–87.1%, respectively [19,20,29]. A number of studies examine factors independently associated with either OS or PFS (or both) in multivariate analyses; these data, when available, are presented in Table 1.

The extent of resection has been consistently associated with lower tumor recurrence rates and longer patient survival in retrospective studies of WHO grade 2 tumors [2,14,39]. As a brain invasion is a common hallmark of these tumors, a meticulous microsurgical technique is required. Several large-scale retrospective examinations of national databases have demonstrated a survival benefit to a greater extent of resection. In an analysis of 575 patients from the Surveillance, Epidemiology, and End Results (SEER) database, Aizer et al. reported 5-year overall survival (OS) rates of 91.3% and 78.2% for patients who underwent GTR and subtotal resection (STR), respectively, and this survival benefit was associated with a hazard ratio (HR) benefit of 0.39 (95% CI, 0.23–0.67; *p* < 0.001) in a multivariate analysis [2]. An examination of 7811 patients culled from the National Cancer Database by Rydzewski and colleagues found a similar benefit of GTR on OS, which was further improved by the addition of RT. The authors found that GTR alone had an HR of 0.71 (95% CI, 0.55–0.92, *p =* 0.009) but that GTR plus RT further improved the HR to 0.47 (*p* = 0.002, 95% CI not reported) [16]. Moon and colleagues studied 55 patients with atypical meningiomas with an average follow-up of 3.8 years [22]. With a GTR rate of 50.9%, they found that the relative risk of recurrence was higher for patients who underwent STR versus GTR (37% versus 14%; *p* = 0.05) regardless of postoperative RT.

A younger age at the time of surgery, better functional status, lower mitotic rate, and no involvement of the adjoining bone have also been associated with a decreased risk of recurrence [20,26]. Da Broi and colleagues examined 77 patients undergoing treatment for WHO grade II meningiomas at a single institution and found that in addition to younger age, higher preoperative KPS was an independent beneficial prognostic indicator. Patients over the age of 65 had an HR of 1.08 (95% CI, 1.04–1.12, *p* < 0.001) for OS, and those with poor KPS (<70) had an HR of 4.00 (95% CI, 1.49–11.11, *p =* 0.006) [29]. Additionally, patients who required any form of retreatment experienced shorter OS than those who did not (HR 2.13, 95% CI, 1.06–4.28, *p* = 0.033) [29].

WHO grade 2 meningiomas tend to have the widest range of prognoses within the three WHO grades. Biological heterogeneity may be contributing to these inconsistent findings, and recent studies have suggested that utilizing molecular criteria would allow for more accurate prognostication [40,41]. Sahm et al. used the unsupervised clustering of DNA methylation profiling to identify three clinically relevant methylation classes regardless of the WHO grade [40]. This stratification provided more precise prognostication of meningioma progression than WHO grading (*p* = 0.096). Interestingly, WHO grade I tumors classified as an intermediate risk of progression using methylation data behaved similar to WHO grade II tumors, whereas WHO grade II tumors classified as a benign risk using methylation data behaved similar to WHO grade I tumors [40]. Nassiri and colleagues have recently integrated molecular data, such as DNA somatic copy-number aberrations, somatic point mutations, methylation, and messenger RNA number, to generate molecular categories of meningiomas that better predicted clinical behavior than traditional grading schemes [42].

Genome-wide analyses of high-grade meningiomas have revealed that these more aggressive tumors harbor more mutations than their lower-grade counterparts [41]. Bi and colleagues compared the genomes of 134 high-grade tumors to their low-grade counterparts and found that high-grade tumors were more likely to possess gene alterations in the NF2 gene as well as more gain and loss mutations, suggesting that a possible driver of high-grade tumors is genomic disruption and NF2 mutations [41].

### 4.3. Adjuvant Treatment

There remains considerable divergence pertaining to the use of adjuvant radiotherapy (RT) following the resection of WHO grade 2 meningiomas. Evidence surrounding the survival benefit that RT confers after either STR or GTR is mixed. In the large retrospective analysis of the National Cancer Database by Rydzewski et al., GTR and RT were both independently but also tandemly associated with longer OS; 5-year OS was highest for patients receiving GTR plus adjuvant RT (HR 0.47, *p* = 0.002) [16]. On the other hand, in a retrospective analysis of 228 patients, Hardesty and colleagues demonstrated a survival benefit of GTR over STR, but adjuvant radiotherapy did not influence PFS for either group of patients (RR for stereotactic radiosurgery 1.0, *p* = 0.99; for intensity-modulated radiotherapy, 0.717, *p* = 0.45) [23].

The effect of radiotherapy on local disease control following GTR is similarly not well established in the literature. Aizer’s study involving 575 patients reported the absence of local recurrence at five years in 82.6% (95% CI, 55.2–94.1%) of patients who received postoperative RT versus 67.8% (95% CI, 50.3–80.2%) in those who did not; this factor was significant in a multivariate model (HR, 0.25; 95% CI, 0.07–0.96; *p =* 0.04) [8]. Park’s analysis of 83 patients treated with an average follow-up of 3.6 years showed improved PFS in patients treated with RT versus those who did not (58.7% versus 44.3% at 5 years, *p =* 0.029). In the authors’ multivariate model, RT and GTR were associated with better PFS. Interestingly, as the authors note, RT had no effect on PFS in the GTR group but did improve PFS in those patients undergoing STR (*p <* 0.001) [24]. Tosefsky and colleagues found that both upfront and delayed RT was associated with improved OS in their retrospective, multi-institutional cohort of 103 patients [38].

Other studies, however, have failed to demonstrate such a recurrence benefit. In the analysis by Hardesty et al., though GTR and adjuvant RT conferred 100% PFS at 73 months follow-up, only 8 patients in their cohort underwent this therapeutic combination, and the result was not statistically significant [23]. Komotar et al. found in their study of 45 patients that 92% of patients with postoperative RT after GTR had no recurrence at a mean 44.1 months, compared to 59% of patients who did experience recurrence without RT, though the result was not significant [43]. Interestingly, Durand et al. found in their series of 166 patients that the addition of RT following the resection of grade II meningiomas decreased PFS. Disease-free progression decreased from 65.7 months without RT to 35.2 months with RT (*p =* 0.0006). However, the authors note that RT was only performed in cases of STR in this cohort and, furthermore, was more frequently performed for recurrences. Their study was underpowered to examine the effects of RT after initial STR [18].

In Rogers et al.’s phase 2 trial investigating RT for “high-risk” meningioma patients (defined as the GTR or STR of new or recurrent WHO grade III tumors, GTR or STR of recurrent WHO grade II tumors, and STR of de novo WHO grade II tumors), RT conferred a 3-year PFS of 58.8% and 3-year OS of 78.6% [44]. The authors found that nearly 93% of recurrences occurred within the previously irradiated field and that recurrent WHO grade II tumors had worse outcomes than newly diagnosed WHO grade III tumors, though the results were not statistically significant.

The question of whether to perform RT following STR remains unanswered; most institutions elect to utilize it after STR but not after GTR. It is important to note the difficulty in extrapolating conclusions from case series, which may employ vastly different radiotherapy protocols. Randomized clinical trials are underway to decipher the optimal management in this clinical scenario [23,31,45,46,47].

The planning of postoperative radiotherapy for recurrent or residual mengiomas may be enhanced by imaging patients with ^68^Ga-DOTATATE in positron emission tomography (PET) scans. While radiosurgical planning typically makes use of MRI scans, the addition of PET scans with ^68^Ga-DOTATATE was found to alter the treatment plans of 5 of 12 patients with either recurrent or newly-diagnosed meningiomas chosen to undergo radiosurgery in Hintz’s study examining the technique. Furthermore, 9 of 12 patients had the disease present on PET scans when it was not appreciable on MRI [48].

### 4.4. Future Developments

To date, retrospective studies analyzing surgical outcomes have relied on the current WHO grading system for their inclusion criteria. The pooling of disparate biological entities within this heterogenous class of tumors may have obscured the possibility of dissimilar effects on tumor behavior and patient survival. Taken together, the effect of microsurgical resection on the natural history of atypical meningioma requires reconsideration in the context of updated molecular classification criteria. A paucity of studies to date have considered molecular characteristics in statistical analyses on survival and outcomes. Further studies in this area will allow for continued refinement in the postoperative management of patients after initial resection as well as for those who present with recurrence.

## 5. WHO Grade 3 Meningiomas

### 5.1. Pathologic Features

Anaplastic (WHO 3) meningiomas are an extremely aggressive class of tumors. They are classified based on a high mitotic index (≥20 mitoses per 10 high powered fields), and the most recent classification scheme attributes the presence of certain molecular features, such as *TERT* promoter methylation and *CDKNA2A/B* loss, as diagnostic for this malignant subtype [49]. Such aggressive tumors account for less than 3% of meningioma diagnoses [12].

### 5.2. Surgical Outcomes and Survival

Maximal resection and conformational radiotherapy are advocated to optimize outcomes, but survival remains poor. The median overall survival (OS) ranges from 1.5 to 3.5 years, and the 5-year OS rate is typically reported to be less than 50% (Table 2) [30,32,33,34,50]. Due to the rarity and aggressive nature of this pathology, single-center retrospective studies are often considered underpowered to study the effects of surgical resection on this disease. However, larger population-based studies have found GTR to be an independent predictor of survival, including analyses of 1936 and 755 patients from the National Cancer Database (NCD) [2,32]. In Rydzewski’s large-scale analysis, GTR in combination with RT conferred a survival benefit, but this result was not statistically significant (HR 0.67, *p =* 0.174) [16]. Champeaux and colleagues studied an impressive 178 patients with anaplastic meningiomas and found a survival benefit with GTR. Age < 65, Simpson I and II resections, and RT following surgery had beneficial effects on OS in a multivariate analysis. Simson I and II resections conferred an HR of 0.51 (95 CI, 0.34–0.78, *p =* 0.0016) [36]. Yang’s analysis of 24 patients with anaplastic meningiomas treated at a single institution found both an OS and PFS benefit to GTR in multivariate modeling (OS HR 2.529, 95% CI, 1.205–5.309, *p <* 0.001; PFS HR 2.12, 95% CI, 1.140–3.949, *p =* 0.018) [19]. The recurrence rate was 75% in the anaplastic group, and secondary anaplastic meningioma patients had a higher risk of death than primary anaplastic meningioma patients (OR 13.507, 95% CI, 2.015–90.556, *p =* 0.007) [19].

Nonetheless, the survival benefit of GTR may be negated if overly aggressive tumor resection leads to permanent neurological morbidity. In a retrospective study of 63 patients with anaplastic meningiomas, patients with near-total resection experienced longer OS than patients with GTR at both initial (*p* = 0.035) and repeat (*p* = 0.005) resection [34]. The authors reported an overall complication rate (including medical complications) of 41% in their cohort, with a neurosurgical complication rate of 21%. There was no difference in either complication statistic between totally and sub-totally resected tumors, and the authors hypothesized that the serious neurological sequelae of these complications may be due to the surgical trauma related to the resection of tumors with pial transgression. It is important to note that in this study, the definition of STR encompasses tumors that were >90% resected; the authors thus concluded that attempting to resect the remaining 1–5% of the tumor in areas with significant operative morbidity may be deleterious.

Similar to the surgical management of aggressive gliomas, it is also important to consider patient health and age prior to the resection of anaplastic meningiomas and in prognostication [51]. As with atypical meningiomas, patients at a younger age at index treatment as well patients with tumors with lower mitosis counts have been found to have improved OS compared to older patients [18,26]. The convexity tumor location has also been implicated as providing improved OS. Palma’s series of 29 patients treated at a single institution found a beneficial effect on OS for tumors located on the convexity in a multivariate model (*p* = 0.0137, HR and CI not reported) [17].

Recently, molecular features have been implicated in the survival of WHO grade 3 meningioma patients. Yang and colleagues found that tumors that overexpressed *p53* had worse prognosis [19,33]. The overexpression of *p53* conferred an HR of 3.019 (95% CI, 1.725–8.625, *p* = 0.034) and 2.878 (95% CI, 1.549–7.818, *p* = 0.026) for OS and PFS, respectively [19]. Additionally, the overexpression of *p53* was found to be an independent risk factor for malignant progression, and malignantly progressed tumors fared far worse overall in their study (OR 5.753, 95% CI, 1.551–21.329, *p* = 0.009). Peyre and co-authors found that *TERT* promoter-mutated tumors fared worse in their study of 57 patients across several institutions but only amongst secondary anaplastic tumors (*p* = 0.02, log rank test) [33].

As detailed above, secondary anaplastic meningiomas (recurrent tumors that progressed from a lower grade) have been associated with worse prognoses than de novo tumors in recent studies [30,33,35]. Variance in the histomolecular characteristics of these tumor types contributes to this difference [33]. There is, however, significant heterogeneity in the clinical course preceding anaplastic transformation in patients with secondary tumors. Specifically, these patients experience varying patterns of relapse, resulting in a multitude of previous surgical, radiotherapeutic, and chemotherapeutic treatment pathways. These differences pose challenges to neurosurgeons and neuro-oncologists and may drive divergent operative and postoperative courses and contribute to disease morbidity. For example, while retrospective studies have advocated for postoperative RT following the resection of de novo anaplastic meningiomas, the utility of radiotherapy following the resection of recurrent high-grade meningiomas is unclear [37,45,47]. Intracranial brachytherapy can be attempted when previous external beam radiation treatment options have been exhausted, but the utility of this treatment modality lacks evidence [52,53].

### 5.3. Adjuvant Treatment

Radiation treatment is often performed for anaplastic tumors regardless of the extent of resection. In Orton’s analysis of the National Cancer database, 52% of patients with anaplastic meningioma underwent postoperative RT, and the authors found a trend towards increased utilization over time as well as geographic variability [32]. The highest rates of utilization were in the Pacific Northwest as well as the West North Central U.S., and the lowest rates occurred in the West South Central regions.

Overall, available retrospective evidence suggests a survival benefit for RT following resection. In Orton’s analysis, RT decreased the risk of death in a multivariate analysis (HR 0.79, *p =* 0.04), although the benefit was not as clear for GTR versus STR (HR for STR 1.57, *p* = 0.02) [32]. Additionally, it is unclear whether the benefit of RT stems from its ability to delay recurrence; Champeaux and colleagues note that while they found a benefit to RT in anaplastic meningiomas, the Kaplan–Meier curves for patients treated with RT versus no RT converge around 7.75 years after treatment [36]. No studies included in the present analysis found a benefit of RT in extending progression-free survival in anaplastic meningiomas.

### 5.4. Future Developments

It was recently suggested that the pre-malignant period of secondary anaplastic patients can predict their prognosis [33]. In 29 patients with secondary anaplastic tumors, Peyre et al. found short time to relapse as a lower-grade meningioma to be associated with OS (<36 months versus >48 months; *p* = 0.0007) [33]. Taken together, these findings stress the need for an improved pathological stratification of meningiomas as well as the need to identify targeted therapies for tumors with genetic profiles conferring aggressive biological behavior.

As with atypical meningiomas, a genomic and molecular analysis of meningiomas may drive prognostication and therapies for anaplastic tumors. A variety of scoring systems incorporating genomic information rather than the simple histopathological grade have been developed [54,55]. These scoring systems, including that developed by Maas and colleagues and that include DNA methylation data as well as copy-number information, have been shown to more accurately predict the risk of recurrence than traditional WHO grading in a cohort of 2868 patients [54]. Driver and colleagues developed a grading scheme consisting of three separate categories, which took into account mitotic count and the loss of certain chromosomes and was found to more accurately identify tumors at risk for recurrence [55]. While these scoring systems are heterogenous and incorporate various genomic features, they must be combined with the extent of resection to guide postoperative treatment and are not widely available due to the cost of sequencing.

## 6. Recurrent High-Grade Meningiomas

Recurrent high-grade meningiomas are a challenging and relatively common problem in neurosurgical oncology. As discussed, even after the GTR of atypical meningiomas, the 5-year progression-free survival (PFS) rate may approach 50% [12,46]. This often necessitates reoperation and re-resection—a difficult task to perform safely while still providing a meaningful improvement in disease-specific outcomes. Dural adhesions to the cortex can lead to the disruption of the pial surface or the tearing of cortical veins [56]. Intradurally, the tumor–arachnoid plane can be difficult or impossible to identify in the presence of scar tissue and radiation-induced changes.

In an analysis of 111 revision resections for non-skull base meningiomas of all WHO grades, Magill et al. reported an overall complication rate of 48% [57]. Tumor location within the middle third of the sagittal plane was found to be associated with perioperative complications. The presence of large venous structures, such as the superior sagittal sinus and Rolandic veins, as well as nearby eloquent cortical anatomy (primary motor cortex) were thought to influence this finding. Importantly, recent efforts to genomically characterize high-grade meningiomas have found an association with paravenous origin (parasagittal, parafalcine, or tocular location) [41]. Thus, recurrent high-grade meningiomas are commonly found in this complex anatomical location.

A recent retrospective analysis of 59 patients with recurrent atypical meningiomas studied the effects of volumetric EOR in revision craniotomy [58]. EOR variables significantly impacted both PFS and OS in a multivariate analysis. With a median follow-up duration of 95 months, GTR (*p* < 0.01) was associated with longer PFS, while a lower Simpson grade (*p* = 0.049) and residual tumor volume (*p* < 0.001) were associated with longer OS. Decreasing residual tumor volumes demonstrated a step-wise increase in patient survival in a Kaplan–Meier analysis, suggesting that even when complete resection is not possible, maximal cytoreduction had a significant impact on clinical outcomes. Ultimately, these results appeared consistent with the clinical practice of performing serial re-resections for maximal cytoreduction in appropriate surgical candidates. Even so, Rubino et al. recently published results adding to the controversy over the management of previously irradiated high-grade meningiomas [47]. In their retrospective cohort of 11 patients with WHO grade II meningiomas and 4 patients with WHO grade III meningiomas who had undergone previous RT, EOR was not associated with PFS after repeat resection, whereas repeat RT after STR in WHO grade II tumors (*p* = 0.003) and repeat RT alone in WHO grade III tumors (*p* = 0.003) was associated with tumor control.

## 7. Future Directions

Genetic and epigenetic profiling efforts have identified meningioma subgroups with diverse clinical and pathological features [40,59,60,61]. Large cohort sequencing has demonstrated that these genomic subgroups often reside in discrete anatomical locations. Historically, the Simpson grading scale has been used by neurosurgeons to predict meningioma recurrence. While still valued today as a surrogate for the extent of resection and as a guide for establishing goals of surgery, this scale has several limitations for predicting long-term tumor control by itself. Most notably, it combines all subtotal resections into a single at-risk group, does not differentiate the residual tumor volumetrically, does not differentiate tumors by intracranial location, and does not account for tumor biology. There is a need for updated, location-specific risk analyses of meningioma progression that incorporate tumor genomics.

In other central nervous system tumors, such as medulloblastoma and low-grade glioma, stratification by molecular subtype has influenced prognoses and led to the investigation of targeted therapies [62,63]. This approach should also be pursued in high-grade meningioma in combination with our continued refinement in microsurgical techniques to resect these challenging tumors.

## 8. Conclusions

Aggressive meningiomas pose surgical and medical challenges to neurosurgeons and neuro-oncologists. Anatomical, clinical, and biological factors influence patient outcomes. Maximal cytoreduction with the preservation of neurological function are the mainstays of surgical management. A versatile skill set with technical mastery are required to optimize surgical results. Future risk analyses of meningioma progression should be multi-faceted and incorporate the extent of resection, residual tumor volume, intracranial location, and tumor biology. Ultimately, these prognostic measures need to offer actionable targets for directed medical therapies in conjunction with successful resection.
